# Preoperative Plasma Cell-Free DNA Integrity Index in Clear Cell Renal Cell Carcinoma: An Exploratory Case–Control Study

**DOI:** 10.3390/cancers18162557

**Published:** 2026-08-09

**Authors:** Tomasz Milecki, Jan Stępka, Joanna Wesoły, Wojciech A. Cieślikowski

**Affiliations:** 1 Department of Urology and Oncological Urology, Poznan University of Medical Sciences, 60-355 Poznań, Poland; 2 Doctoral School, Poznan University of Medical Sciences, 61-701 Poznań, Poland; 3 Department of Urology, Ministry of Internal Affairs Hospital, 61-701 Poznań, Poland; 4 Laboratory of High Throughput Technologies, Faculty of Biology, Institute of Molecular Biology and Biotechnology, Adam Mickiewicz University, 61-614 Poznań, Poland

**Keywords:** circulating tumour DNA, DNA fragmentation, liquid biopsy, kidney cancer, qualitative marker, renal cell carcinoma, cfDNA integrity index

## Abstract

Background/Objectives: Evaluation of renal tumour masses relies on conventional imaging, which cannot reliably characterise the histopathological type, and no validated blood-based diagnostic marker is available for renal cell carcinoma. The cfDNA integrity index, the ratio of long to short circulating cell-free DNA (cfDNA) fragments, may reflect the necrotic origin of tumour-derived DNA and is a candidate qualitative marker of malignancy. This exploratory, single-centre case–control study assessed the preoperative plasma cfDNA integrity index in patients with clear cell renal cell carcinoma (ccRCC). Methods: Plasma concentrations of 90 bp and 222 bp cfDNA fragments were measured by quantitative real-time PCR (qPCR) in 46 patients with histopathologically confirmed ccRCC (before surgery) and 17 healthy volunteers; the two groups were a convenience sample, were not matched, and differed in age and body-mass index. The cfDNA integrity index was defined as the ratio of the 222 bp to the 90 bp fragment concentration. Results: The cfDNA integrity index was higher in patients with ccRCC than in controls (median 0.28 vs. 0.15; *p* < 0.001, Mann–Whitney test) and rose progressively across healthy, non-metastatic (M0) and metastatic (M1) groups (*p* < 0.001, Kruskal–Wallis test; M0 vs. M1 *p* = 0.004). In unadjusted ROC analysis, both the integrity index (AUC 0.83, 95% CI 0.72–0.94) and its long 222 bp fragment component (AUC 0.93) discriminated patients with ccRCC from healthy volunteers, and the index separated metastatic from non-metastatic disease with an AUC of 0.79 (95% CI 0.64–0.88). The index was higher in high-grade (Fuhrman G3 + G4) tumours (*p* = 0.04) and in tumours with lymphovascular invasion (*p* < 0.001), and correlated with primary-tumour diameter. Conclusions: In this exploratory cohort, the preoperative plasma cfDNA integrity index was higher in patients with ccRCC than in healthy volunteers and was associated with several adverse pathological features; these findings require confirmation in larger, matched studies that include benign renal masses before any diagnostic role can be claimed.

## Simple Summary

Renal cell carcinoma is challenging to diagnose non-invasively: imaging cannot reliably distinguish malignant from benign renal masses, and no validated blood biomarker exists. Liquid biopsy (analysis of tumour-derived molecules in blood) offers a promising approach. This study evaluates a simple blood-based parameter, the cell-free DNA (cfDNA) integrity index, which reflects how fragmented circulating DNA is in the bloodstream. In 46 patients with clear cell renal cell carcinoma and 17 healthy volunteers, we show that the cfDNA integrity index is significantly elevated before surgery in patients with cancer, increases with the presence of metastases, and is associated with high-grade tumours and lymphovascular invasion.

## 1. Introduction

Renal cell carcinoma (RCC) accounts for approximately 5% and 3% of all malignancies in men and women, respectively, and clear cell RCC (ccRCC) is the most common subtype, representing about 80% of cases [1,2]. Computed tomography and magnetic resonance imaging are the modalities of choice for evaluating renal tumour masses but have limited ability to determine the histopathological subtype [3]. Distinguishing malignant RCC from benign lesions such as oncocytoma or fat-poor angiomyolipoma (which often do not require surgery) therefore remains an important clinical challenge, particularly for small renal masses, up to 20% of which are benign on final pathology [4]. Two distinct questions therefore arise: whether a blood-based marker can separate patients with cancer from cancer-free individuals, and whether it can separate malignant from benign renal masses.

Liquid biopsy, the analysis of tumour-derived molecules released into body fluids, is being actively investigated to address these limitations [5,6,7]. Among its variants, the analysis of circulating cell-free DNA (cfDNA) is of particular interest. cfDNA is released into the circulation mainly through apoptosis and necrosis [8]. DNA released by apoptosis is cleaved enzymatically into short, uniform fragments of roughly 180–200 bp, whereas DNA released through the necrosis that predominates in malignant tissue circulates as fragments of heterogeneous and frequently greater length [9,10,11]. The cfDNA integrity index, defined as the ratio of the concentration of long to short cfDNA fragments, captures this difference and has shown diagnostic value in several malignancies [12,13]. More recent fragmentomic work indicates that cfDNA fragment-length patterns are shaped by a more complex interplay of nucleosome positioning, chromatin structure and nuclease activity than a simple apoptosis-versus-necrosis dichotomy, so fragment length is best regarded as an empirical readout rather than a direct measure of a single release mechanism [9,10,11].

We have previously reported, in the same prospectively collected cohort, that the preoperative plasma concentration of total cfDNA (the 90 bp fragment) is elevated in ccRCC, increases with stage, and carries independent information on cancer-specific survival [14]. That work addressed the quantitative dimension of cfDNA. Whether the qualitative dimension (the fragment-length distribution captured by the integrity index) adds diagnostic information in ccRCC has been examined in only a small number of studies, with discordant results [15,16,17]. The aim of the present exploratory study was to assess the performance of the preoperative plasma cfDNA integrity index for distinguishing patients with ccRCC from healthy individuals, and its relationship with established pathological prognostic features.

## 2. Materials and Methods

### 2.1. Study Population

The study included 46 patients with histopathologically confirmed ccRCC, consecutively enrolled among patients with a renal tumour mass identified on computed tomography or magnetic resonance imaging and referred for surgery at the Department of Urology and Urological Oncology of the Poznan University of Medical Sciences between 2018 and 2019, together with 17 healthy individuals who constituted the control group (Table 1). Controls were recruited contemporaneously at the same centre from volunteers and patients of the urology department who had no renal mass and no known malignancy, and their blood samples were processed with the identical pre-analytical protocol used for the patients. All participants were informed about the rationale of the project and provided written informed consent. The Bioethics Committee of the Poznan University of Medical Sciences approved the project (Resolution No. 568/17).

Exclusion criteria, verified from the medical history and physical examination, were poor general condition (ECOG performance status >1), inflammatory disease, autoimmune disease, renal failure, and the presence of any other malignancy. As part of the preoperative work-up, every patient underwent a structured history and physical examination, anaesthetic assessment, urinary-tract ultrasonography, routine laboratory tests (complete blood count, coagulation panel, and serum glucose, electrolyte and creatinine measurements), and clinical staging by contrast-enhanced abdominal and pelvic computed tomography together with chest imaging. Controls were evaluated by the same history and physical examination but did not undergo additional laboratory screening to exclude subclinical renal, inflammatory or malignant disease. The two groups were a convenience sample, were not matched for age, sex or body-mass index, and no formal a priori sample-size calculation was performed; all eligible patients with ccRCC operated on during the study period were included.

### 2.2. Material Collection

Before surgery, 9 mL of peripheral blood was collected from each participant into K2EDTA tubes. Samples were transported to the laboratory on the day of collection and processed immediately on arrival, so that the interval between venepuncture and plasma separation did not exceed 24 h. To separate plasma, the blood was centrifuged once at 400 *g* for 30 min at room temperature; this low-speed step was chosen to sediment the cellular fraction and recover plasma while limiting mechanical disruption of leucocytes. The plasma was then aliquoted and stored at −80 °C until batched analysis. The identical standardised pre-analytical protocol (fixed blood volume, EDTA collection tubes, single centrifugation at 400 *g* for 30 min at room temperature immediately after transport, aliquoting, and storage at −80 °C) was applied to all participants in both groups to minimise pre-analytical variability. Each aliquot underwent a single freeze–thaw cycle before cfDNA extraction.

### 2.3. Extraction of Cell-Free DNA

cfDNA was extracted from plasma using the spin-column method with the QIAamp^®^ Circulating Nucleic Acid Kit (QIAGEN, Hilden, Germany), following the manufacturer’s protocol. After thawing, 3 mL of plasma was centrifuged (14,000 rpm, 10 min) at room temperature before extraction, and cfDNA was eluted in 25 µL of AVE buffer and stored at −20 °C until analysis. The concentration of the eluted cfDNA was measured fluorometrically (Qubit 2.0 Fluorometer with the dsDNA HS Assay Kit; Thermo Fisher Scientific, Waltham, MA, USA), and the fragment-size distribution of representative eluates was verified on an Agilent DNA 1200 assay (Agilent Technologies, Santa Clara, CA, USA). To assess reproducibility, the entire procedure (re-extraction followed by re-quantification and re-amplification) was repeated on 12 randomly selected samples, corresponding to approximately 15% of the series. Extraction blanks, no-template negative controls and exogenous spike-in controls were not included, and samples were not processed in randomised batches; a contribution of batch effects to the between-group differences therefore cannot be formally excluded.

### 2.4. Assessment of cfDNA Fragments by Quantitative Real-Time PCR

Concentrations of the 90 bp (short) and 222 bp (long) cfDNA fragments were measured by quantitative real-time PCR (qPCR) with primers complementary to the L1PA2 transposon, a subfamily of LINE (long interspersed nuclear element) sequences that account for about 17% of the human genome and are dispersed throughout nuclear DNA, enabling reliable quantification of cfDNA [18]. The cfDNA integrity index was defined as the ratio of the 222 bp to the 90 bp fragment concentration; because amplification of the 90 bp fragment also captures longer molecules, the index is expected to be <1 and to increase when the proportion of long, necrosis-derived fragments rises.

qPCR was performed with SYBR Green dye (VWR, Radnor, PA, USA) on a CFX96 Connect Real-Time PCR Detection System (Bio-Rad, Hercules, CA, USA) in 48-well plates (Cytogen, Warsaw, Poland). Primers were designed with Primer3 [19]. The L1PA2-90 primers were: forward 5′-TGCCGCAATAAACATACGTG-3′ and reverse 5′-GACCCAGCCATCCCATTAC-3′; the L1PA2-222 primers were: forward 5′-TGCCGCAATAAACATACGTG-3′ and reverse 5′-AACAACAGGTGCTGGAGAGG-3′. Each 10 µL reaction contained 1.2 µL primer mix, 5 µL SYBR, 1.3 µL water and 2.5 µL DNA template. Cycling conditions are given in Table 2.

A four-point standard curve, prepared from genomic DNA of healthy controls quantified spectrophotometrically (NanoDrop, Thermo Fisher Scientific, Waltham, MA, USA) and serially diluted (1:10, 1:100, 1:1000 and 1:10,000), was run in duplicate on every plate and used for absolute quantification from the Ct values and for monitoring amplification efficiency and linearity (R^2^) on each run. Product specificity was confirmed by melt-curve (dissociation) analysis at the end of every run. Each sample was analysed in two technical replicates, and full biological replicates (re-extraction and re-amplification) were performed on 12 randomly selected samples, approximately 15% of the series. A formal analytical validation of the assay, including determination of the limits of detection and quantification and of intra- and inter-assay precision, was not performed, so the quantitative estimates should be regarded as exploratory.

### 2.5. Statistical Analysis

Continuous variables were compared with the independent-samples *t*-test or the Mann–Whitney U test, and categorical variables with the χ^2^ test, according to distribution (Shapiro–Wilk test). The Kruskal–Wallis test with Dunn’s multiple-comparison correction was used for comparisons across three groups. Diagnostic performance was assessed by receiver-operating-characteristic (ROC) analysis; the area under the curve (AUC) was calculated and optimal cut-off points were identified with Youden’s index. Correlation with tumour diameter was assessed with Spearman’s coefficient. The following confirmatory analyses were also performed: a multivariable logistic-regression model of ccRCC status adjusting for age, body-mass index and sex (continuous predictors standardised per 1 SD); correction of the pathological-subgroup comparisons for multiple testing by the Benjamini–Hochberg false-discovery-rate (FDR) method; and Cox proportional-hazards and Kaplan–Meier analysis of cancer-specific survival. A two-sided *p* < 0.05 was considered statistically significant. Analyses were performed with IBM SPSS Statistics (v25; IBM Corp., Armonk, NY, USA) and Statistica (TIBCO Software 10.4, Palo Alto, CA, USA).

## 3. Results

### 3.1. cfDNA Integrity Index Discriminates ccRCC from Healthy Individuals

**Figure 1:**
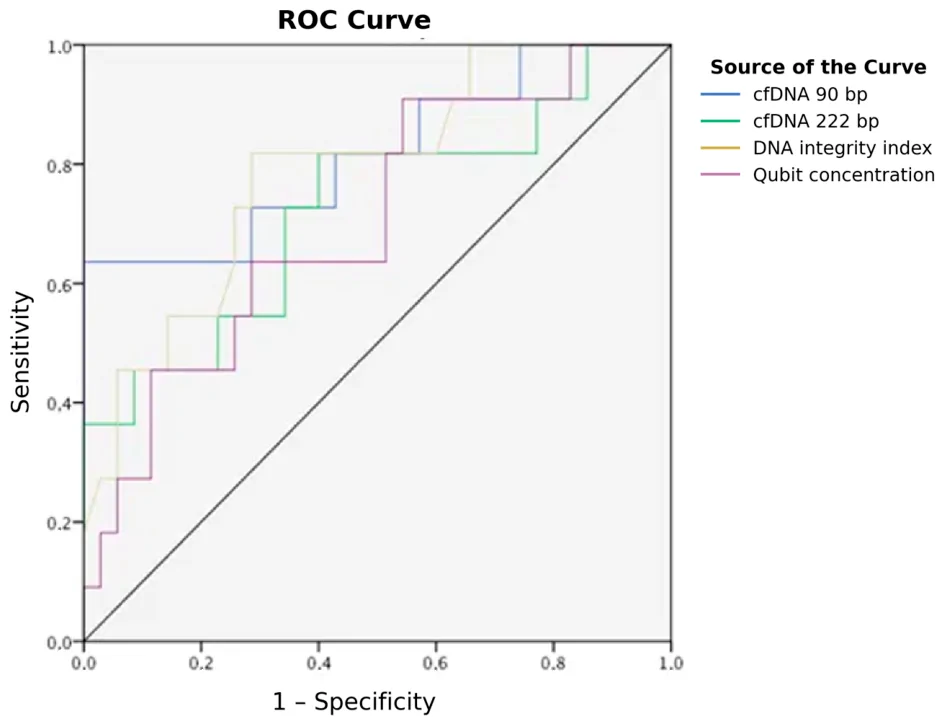
ROC curves for distinguishing patients with ccRCC from healthy controls using the cfDNA integrity index, the 222 bp fragment and the 90 bp fragment.

The cfDNA integrity index was significantly higher in patients with ccRCC than in healthy controls (median 0.28 vs. 0.15; *p* < 0.001, Mann–Whitney test). The increase reflected a disproportionate rise in the long (222 bp) fragment: both the 90 bp and the 222 bp fractions were higher in patients with ccRCC than in controls, but the relative increase was greater for the long fragment (Table 3). In ROC analysis, the integrity index distinguished patients with ccRCC from controls with an AUC of 0.83 (95% CI 0.72–0.94; cut-off 0.20; sensitivity 80%, specificity 65%); the long 222 bp fragment alone performed best (AUC 0.93) (Table 4, Figure 1). After adjustment for age, body-mass index and sex in a multivariable logistic-regression model, the integrity index remained independently associated with ccRCC (OR 5.72 per 1 SD, 95% CI 2.13–15.39, *p* = 0.001).

### 3.2. The Integrity Index Increases with Metastatic Stage

**Figure 2:**
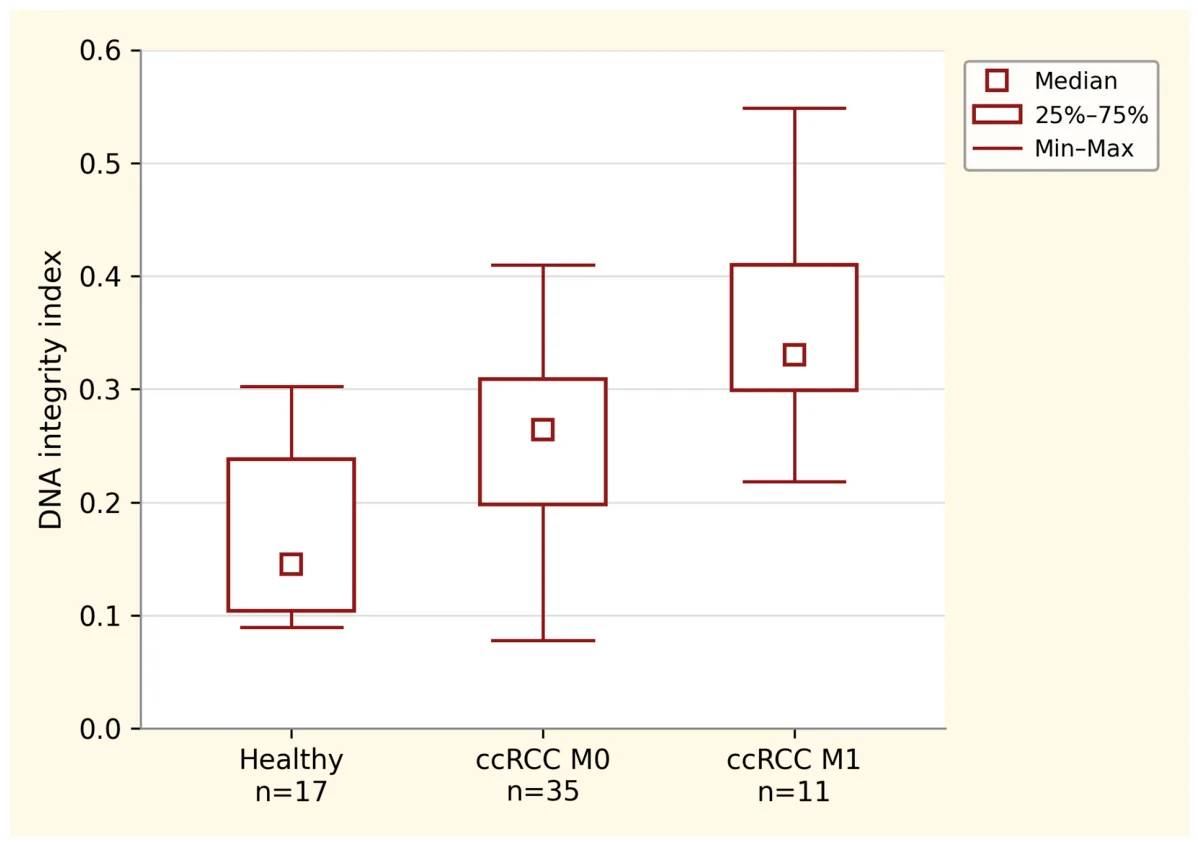
Plasma cfDNA integrity index (222 bp/90 bp) in healthy controls and in patients with non-metastatic (M0) and metastatic (M1) ccRCC. Boxes show the interquartile range, the horizontal line the median, the square the mean, and whiskers the range.

**Figure 3:**
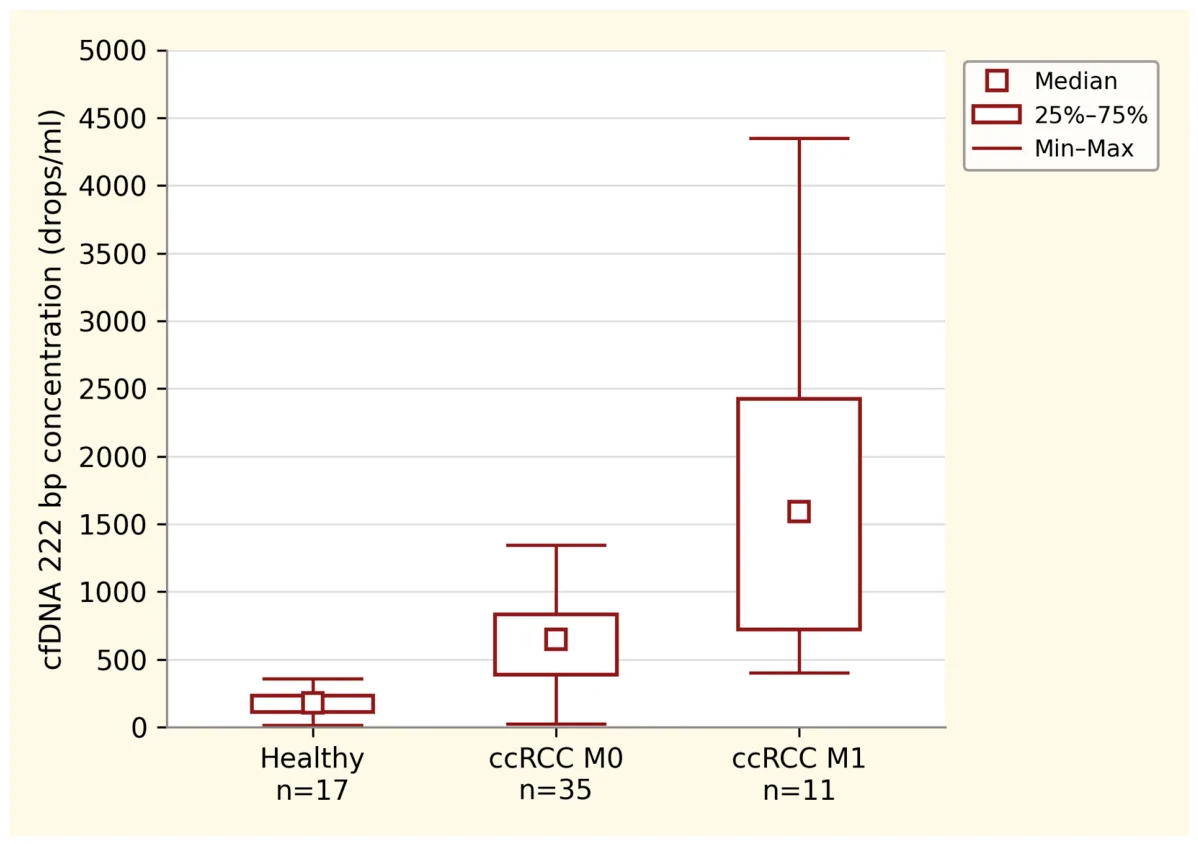
Plasma concentration of the long (222 bp) cfDNA fragment in healthy controls and in patients with non-metastatic (M0) and metastatic (M1) ccRCC.

**Figure 4:**
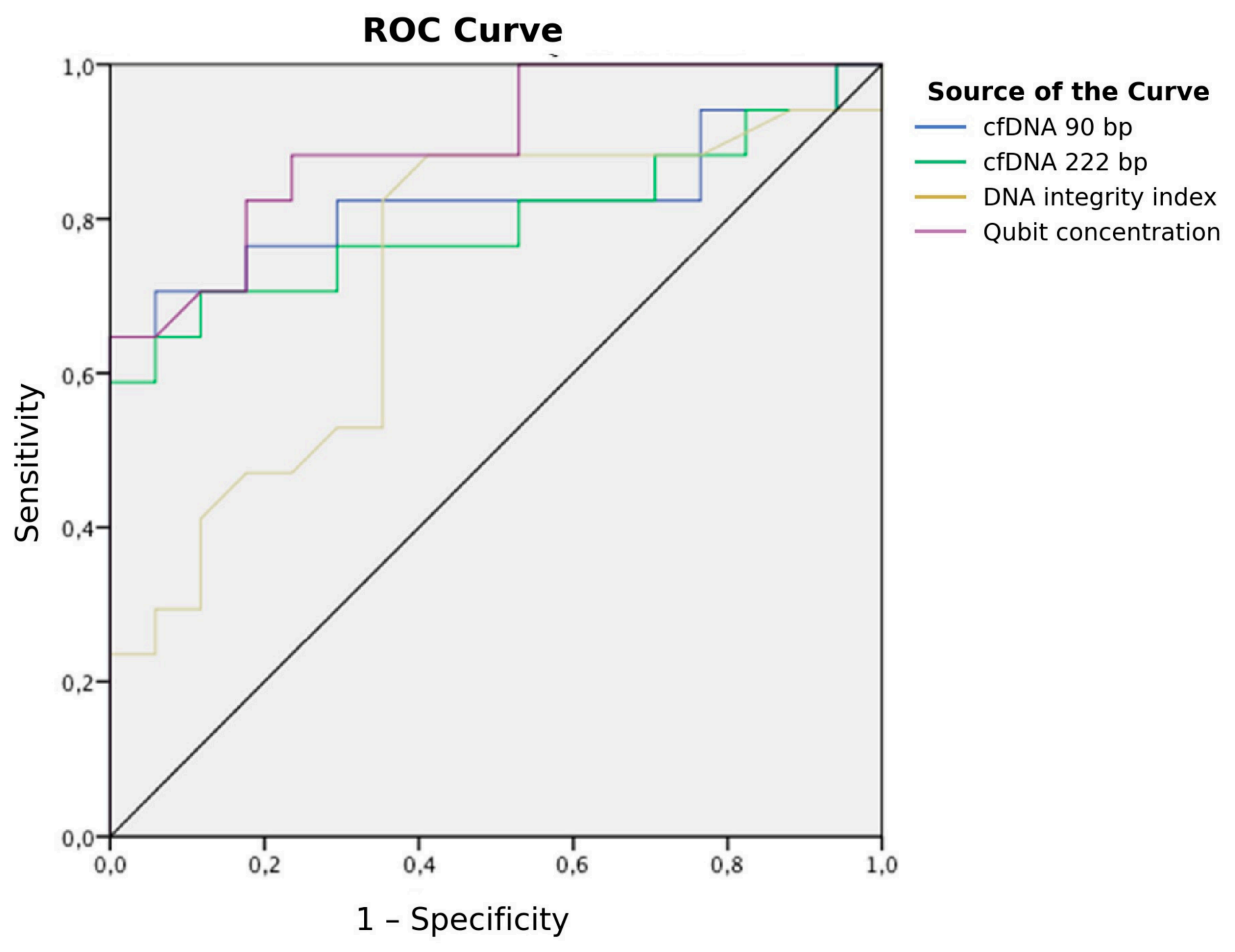
ROC curves for distinguishing metastatic (M1) from non-metastatic (M0) ccRCC.

The integrity index rose progressively across the healthy, M0 and M1 groups, with a significant overall difference (*p* < 0.001, Kruskal–Wallis test) and significant pairwise differences (healthy vs. M0 and healthy vs. M1, both *p* < 0.005; M0 vs. M1, *p* = 0.004) (Table 3, Figure 2). The long 222 bp fragment showed the same stage-dependent pattern (Figure 3). The integrity index distinguished metastatic (M1) from non-metastatic (M0) disease with an AUC of 0.79 (95% CI 0.64–0.88; cut-off 0.30; sensitivity 73%, specificity 71%) (Table 5, Figure 4).

### 3.3. The Integrity Index Reflects Adverse Pathological Features

Within the ccRCC group, the integrity index was significantly higher in high-grade tumours (Fuhrman G3 + G4, n = 16) than in low-grade tumours (G1 + G2, n = 30) (*p* = 0.04, Mann–Whitney test) and in tumours with lymphovascular invasion (n = 13) than in those without (n = 33) (*p* < 0.001). The index correlated positively with the diameter of the primary tumour (Spearman ρ = 0.30, *p* = 0.04). Thus, the index tracked the principal established adverse pathological features of ccRCC. After Benjamini–Hochberg correction for multiple testing, the associations with lymphovascular invasion (FDR-adjusted *p* = 0.002), metastatic stage (FDR-adjusted *p* = 0.009), Fuhrman grade (FDR-adjusted *p* = 0.04) and tumour diameter (FDR-adjusted *p* = 0.04) all remained significant; given the small subgroups, these are nonetheless best regarded as exploratory.

### 3.4. Discrimination of Early-Stage Disease

In the subgroup of patients with clinical stage I ccRCC (n = 17), all studied parameters were significantly higher than in controls. The integrity index distinguished patients with stage I ccRCC from healthy individuals with an AUC of 0.73 (95% CI 0.55–0.90; sensitivity 82%, specificity 65%), indicating that the qualitative change in cfDNA is detectable even in early disease.

**Figure 5:**
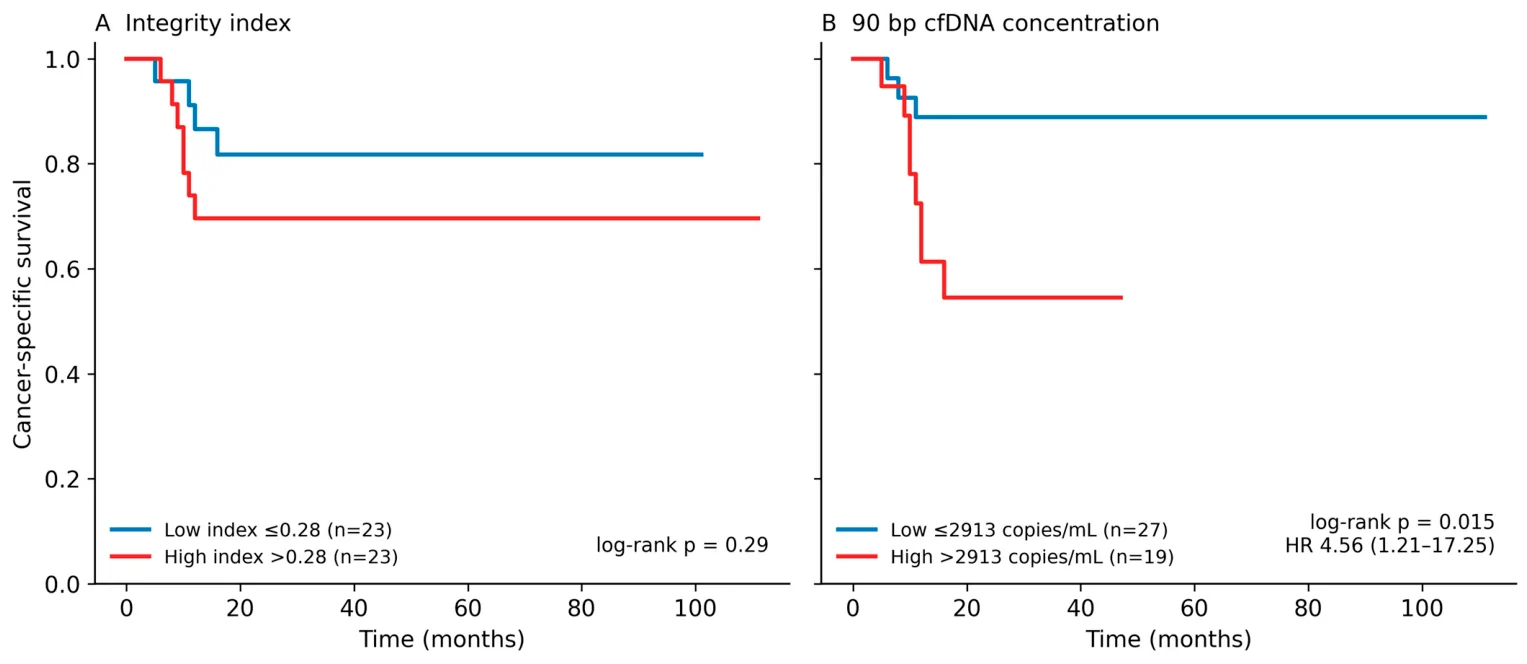
Kaplan–Meier estimates of cancer-specific survival in patients with ccRCC stratified by (**A**) the cfDNA integrity index (median split; log-rank *p* = 0.29) and (**B**) the 90 bp cfDNA fragment concentration (cut-off 2913 copies/mL; log-rank *p* = 0.015; HR 4.56, 95% CI 1.21–17.25). The prognostic signal is carried by cfDNA quantity, not by the fragment-length ratio.

A prognostic analysis of the integrity index was also performed in the ccRCC group; it did not discriminate between patients who died of disease and survivors (AUC 0.58, not significant) and was not retained in survival modelling. Over a median follow-up of 22 months (range 5–111) with 11 cancer-specific deaths, the integrity index was not associated with cancer-specific survival (HR 1.30 per 1 SD, 95% CI 0.75–2.27, *p* = 0.36; prognostic AUC 0.58). By contrast, and consistent with our earlier report [14], the 90 bp fragment concentration was strongly prognostic (HR 2.79 per 1 SD, 95% CI 1.71–4.56, *p* < 0.001; dichotomised at 2913 copies/mL, HR 4.56, 95% CI 1.21–17.25, log-rank *p* = 0.015) (Figure 5).

## 4. Discussion

In this study, the preoperative plasma cfDNA integrity index was significantly higher in patients with ccRCC than in healthy individuals, increased with metastatic stage, and was associated with high Fuhrman grade, lymphovascular invasion and larger tumour diameter. The increase in the index was driven by a disproportionate rise in long cfDNA fragments, consistent with the hypothesis that malignant cells release longer, necrosis-derived DNA into the circulation [9,20].

Three previous studies have evaluated the cfDNA integrity index in RCC, with which our results can be compared. Hauser et al. studied 34 patients with RCC and 54 controls using ACTB-based primers (ACTB-384/ACTB-106) and reported a significantly higher integrity index in patients than in controls (1.07 vs. 0.72, *p* = 0.04), with two- to three-fold higher concentrations of both short and long fragments [15]. Despite the different primers, those findings agree with ours. Gang et al. assessed 78 patients with RCC and 42 controls and measured fragments of 109, 193, 397 and 456 bp [16]. All fragment lengths were detectable before surgery in patients, whereas only the shorter fragments were detected in controls; the integrity index was elevated in patients and increased with stage, and the long fragments became less detectable after surgery, supporting their necrotic, tumour-derived origin. Unlike our study, Gang et al. found no association with Fuhrman grade.

By contrast, Lu et al. measured seven fragments of 67–306 bp in 145 patients with localised and 84 patients with metastatic ccRCC and 40 controls and found that the concentration of short fragments rose with stage, which paradoxically lowered their integrity index [17]. This discrepancy with our results, and with those of Hauser and Gang, most likely reflects measurement of different (and substantially shorter) cfDNA fractions; a decreasing integrity index has also been described in other tumours when very short fragments are measured. Importantly, by combining cfDNA quantity and integrity, Lu et al. still built diagnostic models that separated their groups (AUC 0.84, sensitivity 70%, specificity 88%), and theirs remains the only study to date to report a prognostic role for the integrity index. In our cohort, the integrity index did not carry independent prognostic information, a difference plausibly explained by the smaller sample, shorter follow-up, the inclusion of metastatic patients and the different cfDNA fractions analysed. It must be emphasised that these cross-study comparisons are only qualitative: because the integrity index is defined by the specific primer targets and fragment lengths chosen (L1PA2 222/90 bp here, ACTB 384/106 bp in Hauser et al., 109–456 bp in Gang et al., and 67–306 bp in Lu et al.), absolute index values are not directly comparable across studies. The divergent trends, particularly the inverse behaviour reported by Lu et al., are most parsimoniously explained by the measurement of different and, in that case, substantially shorter cfDNA fractions rather than by a genuine biological contradiction.

Since these early integrity-index studies, renal cell carcinoma liquid-biopsy research has increasingly turned to sequencing-based approaches, including circulating tumour DNA mutational and fragment-size profiling [21,22], cell-free DNA methylation classifiers that detect RCC across all stages with high accuracy [23], and tumour-informed circulating tumour DNA for prognosis and minimal residual disease after nephrectomy [24]. These advances underscore the broader diagnostic value of cfDNA fragmentation analysis in RCC and place the integrity index (validated here using a straightforward, widely available qPCR assay) within an expanding landscape of fragment-length-based liquid-biopsy approaches. Beyond liquid biopsy, tissue-based molecular biomarkers continue to inform ccRCC prognosis and treatment: a meta-analysis of 3389 patients showed that PD-L1 overexpression is associated with worse overall, progression-free and cancer-specific survival and with more advanced clinicopathological features, whereas PD-L2 carries only weak prognostic value [25], and integrative transcriptomic analyses have nominated immune- and therapy-related hub genes (such as CRYBB1, CEACAM4 and HAMP) as candidate biomarkers linked to sunitinib resistance and immune infiltration in ccRCC [26]. Against this background, a minimally invasive qualitative marker such as the plasma cfDNA integrity index could complement, rather than replace, these tissue-based and sequencing approaches.

A notable feature of the present study is the parallel measurement of the 90 bp and 222 bp L1PA2 fractions in a single, prospectively collected cohort, which allowed the quantitative and qualitative dimensions of cfDNA to be analysed with the same assay and demonstrated that both carry independent diagnostic information in ccRCC. As a ratio normalised for total cfDNA input, the integrity index provides a single, directly interpretable measure of fragmentation that is inherently robust to inter-sample variation in extraction yield; in this cohort, it discriminated ccRCC from controls with an AUC of 0.83, while its long 222 bp fragment component reached 0.93. The index thus complements, rather than replaces, quantitative cfDNA measurement.

Several limitations should be acknowledged, most of which are inherent to cfDNA analysis. cfDNA measurements are highly susceptible to pre-analytical variables, including the time interval between blood collection and plasma separation, storage temperature and duration, collection-tube type, and DNA extraction method [27,28,29,30,31]. Plasma is preferred over serum because leucocyte lysis during serum preparation may lead to artificially elevated cfDNA concentrations [32], while the spin-column extraction method used in our study represents the most widely applied approach [33]. qPCR quantifies total cfDNA, including both tumour-derived and non-tumour-derived fractions, and the lack of a universally standardised target sequence limits comparability between studies. Moreover, cfDNA levels may be increased in non-oncological conditions, such as recent myocardial infarction, physical trauma, and systemic diseases [34,35,36]; although these factors were addressed through our exclusion criteria, residual confounding cannot be completely excluded. The control group consisted of healthy individuals rather than patients with benign renal masses; therefore, the ability of the proposed index to distinguish between benign and malignant renal lesions, which represents the key clinical challenge, could not be assessed and requires dedicated investigation. Furthermore, the single-centre design and relatively limited sample size, particularly within the metastatic subgroup, highlight the need for validation in larger, multi-centre cohorts. Additional methodological limitations should also be considered. The ccRCC and control groups differed in age and body-mass index, both of which may influence cfDNA concentration and fragmentation patterns; however, multivariable analysis adjusting for age, body-mass index, and sex confirmed that the integrity index remained independently associated with ccRCC. Nevertheless, validation in an independent cohort is necessary before its clinical applicability can be established. In addition, plasma separation was performed using a single low-speed centrifugation step (400 *g*), and samples underwent a freeze–thaw cycle before extraction; since leucocyte lysis may release longer genomic DNA fragments and consequently increase the long-fragment fraction, the potential contribution of pre-analytical cellular DNA contamination to the integrity index cannot be fully excluded. Importantly, in this cohort, the integrity index did not demonstrate superior performance compared with the 222 bp fragment measurement alone, and therefore its incremental diagnostic value remains unproven All diagnostic cut-offs were derived within the same cohort using Youden’s index without internal validation, raising the possibility of overfitting and limiting generalisability. The qPCR assay also lacked formal analytical validation, including assessment of limits of detection and quantification, intra- and inter-assay precision, as well as extraction and no-template controls. Consequently, the reported findings should be considered exploratory and require confirmation in independent, adequately powered studies.

## 5. Conclusions

In this exploratory single-centre cohort, the preoperative plasma cfDNA integrity index was higher in patients with ccRCC than in healthy controls and was associated with several adverse pathological features, and it could be detected even in early-stage disease. Larger studies incorporating matched controls, benign renal masses, standardised pre-analytics, multivariable adjustment and independent validation are needed to determine its clinical diagnostic value.

## Figures and Tables

**Table 1:** Clinical characteristics of the study participants.

Characteristic	ccRCC (*n* = 46)	Control (*n* = 17)	*p*-Value
Age, years (mean ± SD)	60 ± 8.5	52 ± 17.9	0.012
Sex, male/female	34/12	7/10	0.35
BMI (mean)	28.1	25.5	0.036
Hypertension, n	30	7	0.15
Diabetes mellitus (type 1 + 2), n	8	4	0.70
Smokers (current/former), n	19	4	0.14
Type of surgery, radical/nephron-sparing	38/8	–	–
Pathological stage, pT1/pT2/pT3/pT4	21/3/19/3	–	–
Metastasis, M0/M1	35/11	–	–
Fuhrman grade, low (G1 + G2)/high (G3 + G4)	30/16	–	–
Lymphovascular invasion, absent/present	33/13	–	–
Symptomatic at presentation, n	21	–	–

**Table 2:** Real-time PCR cycling conditions.

Stage	Cycles	Time	Temperature
Polymerase activation	1	10 min	95 °C
Denaturation	40	10 s	95 °C
Annealing/extension		60 s	60 °C
Melt-curve (3 steps)	1	15 s each	95/55/95 °C

**Table 3:** Plasma concentrations of the 90 bp and 222 bp cfDNA fractions and the cfDNA integrity index by group, expressed as median; mean (SD).

Group	90 bp (Copies/mL)	222 bp (Copies/mL)	Integrity Index
Healthy (n = 17)	960; 931 (490)	140; 151 (94)	0.15; 0.17 (0.07)
ccRCC M0 (n = 35)	2473; 2510 (1378)	631; 624 (325)	0.26; 0.26 (0.07)
ccRCC M1 (n = 11)	3619; 5282 (4059)	1584; 1730 (1195)	0.33; 0.36 (0.09)

**Table 4:** Diagnostic performance of the studied parameters for distinguishing patients with ccRCC from healthy controls.

Parameter	AUC (95% CI)	*p*	Cut-Off	Sensitivity (%)	Specificity (%)
222 bp cfDNA	0.93 (0.87–1.00)	<0.001	269 copies/mL	89	88
90 bp cfDNA	0.89 (0.81–0.97)	<0.001	1433 copies/mL	83	88
Integrity index	0.83 (0.72–0.94)	<0.001	0.20	80	65

**Table 5:** Diagnostic performance for distinguishing metastatic (M1) from non-metastatic (M0) ccRCC.

Parameter	AUC (95% CI)	*p*	Cut-Off	Sensitivity (%)	Specificity (%)
222 bp cfDNA	0.82 (0.65–0.98)	0.002	796 copies/mL	73	71
Integrity index	0.79 (0.64–0.88)	0.004	0.30	73	71
90 bp cfDNA	0.73 (0.54–0.91)	0.026	2818 copies/mL	73	66

## Data Availability

The data supporting the findings are available from the corresponding author on reasonable request.
